# A Deep Learning Approach for Segmentation of Red Blood Cell Images and Malaria Detection

**DOI:** 10.3390/e22060657

**Published:** 2020-06-13

**Authors:** Maria Delgado-Ortet, Angel Molina, Santiago Alférez, José Rodellar, Anna Merino

**Affiliations:** 1 Core Laboratory, Biochemistry and Molecular Genetics, Biomedical Diagnostic Center, Hospital Clínic of Barcelona, 08036 Barcelona, Spain; amolinab@clinic.cat; 2 Applied Mathematics and Computer Science, School of Engineering, Science and Technology, Universidad del Rosario, Bogotá 111711, Colombia; edwin.alferez@urosario.edu.co; 3 Department of Mathematics, Technical University of Catalonia, 08019 Barcelona, Spain; jose.rodellar@upc.edu

**Keywords:** deep learning, malaria detection, red blood cell (RBC) segmentation, blood cell classification, convolutional neural networks

## Abstract

Malaria is an endemic life-threating disease caused by the unicellular protozoan parasites of the genus *Plasmodium*. Confirming the presence of parasites early in all malaria cases ensures species-specific antimalarial treatment, reducing the mortality rate, and points to other illnesses in negative cases. However, the gold standard remains the light microscopy of May-Grünwald–Giemsa (MGG)-stained thin and thick peripheral blood (PB) films. This is a time-consuming procedure, dependent on a pathologist’s skills, meaning that healthcare providers may encounter difficulty in diagnosing malaria in places where it is not endemic. This work presents a novel three-stage pipeline to (1) segment erythrocytes, (2) crop and mask them, and (3) classify them into malaria infected or not. The first and third steps involved the design, training, validation and testing of a Segmentation Neural Network and a Convolutional Neural Network from scratch using a Graphic Processing Unit. Segmentation achieved a global accuracy of 93.72% over the test set and the specificity for malaria detection in red blood cells (RBCs) was 87.04%. This work shows the potential that deep learning has in the digital pathology field and opens the way for future improvements, as well as for broadening the use of the created networks.

## 1. Introduction

In 2018, an estimated 228 million cases of malaria occurred worldwide, causing 405,000 deaths according to the World Health Organization (WHO) [1]. Malaria is consequently one of the major global public health challenges and a life-threatening disease [2,3]. It is a parasitic disease caused by the unicellular protozoan parasites of the genus *Plasmodium*. Of more than 120 *Plasmodium* species infecting mammals, birds, and reptiles, only six are known to infect human beings regularly: *P. vivax*, *P. falciparum*, *P. malariae*, *P. ovale curtisi*, *P. ovale wallkeri*, and *P. knowlesi* [4]. *P. falciparum* is the most prevalent malaria parasite, causing 99.7% of estimated malaria cases in Africa in 2018 [1], as well as the cause of the most serious and sometimes fatal type of malaria [5]. *Plasmodium* parasites are transmitted to people through the bites of infected female *Anopheles* mosquitoes, called “malaria vectors”. The risk of transmission exists in over 100 countries and territories in both tropical and subtropical areas [2,6]. These areas are yearly visited by over 125 million international travelers [2], and malaria is consequently imported to non-endemic areas like Europe. Between 120 and 180 cases are registered annually in Spain [6].

The *Plasmodium* parasite’s complex lifecycle involves two hosts: an insect vector (mosquito) and a vertebrate host (human) [7]. When sporozoites (the infective stage of *Plasmodium*) are inoculated into the human bloodstream, the exoerythrocytic cycle begins. In this stage, the parasite replicates itself in the liver cells and releases merozoites. The rupture of hepatic cells determines the beginning of the erythrocytic cycle. Erythrocytes or red blood cells (RBCs) are the most abundant cell in the blood, accounting for about 40–45% of its volume. The other two cellular components suspended in blood are platelets and white blood cells (WBCs). Inside erythrocytes, parasites firstly adopt a ring shape, becoming trophozoites. They then replicate themselves inside the RBC and become schizonts, the accumulation of which will cause the cell to explode. They are then released and infect other erythrocytes, in which they repeat the erythrocytic cycle [6].

**Figure 1:**
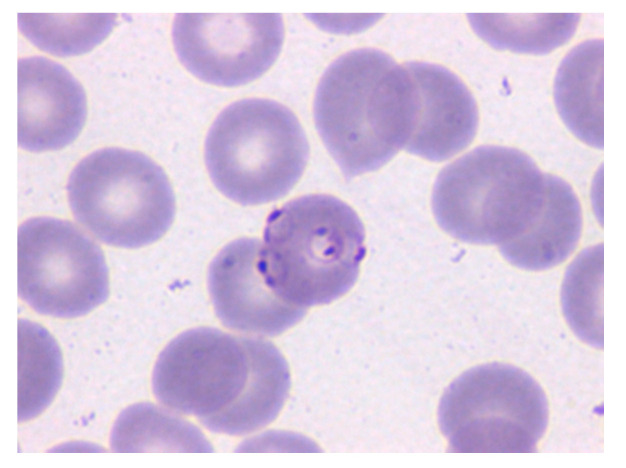
Microscopy image of a May-Grünwald–Giemsa (MGG)-stained peripheral blood (PB) smear in which the erythrocyte in the center is parasitized by *P. Falciparum* (Merino, 2019 p.66). Reprinted with permission.

Due to malaria’s high burden on global health, an estimated $2.7 billion were invested and available for its research in 2018 [1]. However, the gold standard remains light microscopy of the May-Grünwald–Giemsa (MGG)-stained thin and thick peripheral blood (PB) films [3], as the disease is diagnosed into the erythrocytic cycle, given that all the stages in such are visible under light microscope using PB whole-slide smears [7] (Figure 1). The guidelines indicate that parasite quantification in thin films should examine a minimum of 1000 RBCs from different areas of the film and be performed by two trained observers to confirm the diagnosis and species [8]. Therefore, this is a time-consuming procedure that requires pathologists to observe each patient’s samples for between 30 and 60 min [5]. Confirming the presence of parasites early in all malaria cases ensures species-specific antimalarial treatment, reducing the mortality rate [7], and points to other illnesses in negative cases [4]. Moreover, it is dependent on the pathologist’s skills, subjective and error-prone, [9] as it demands a high level of expertise and healthcare providers may encounter difficulty in diagnosing malaria in places where it is not endemic [3,5].

In the context of the morphological analysis of cells circulating in blood, machine learning (ML) methods have been proposed to achieve higher imaging diagnostic precision [7,10]. Recently, Deep Learning (DL) has gained attention since it allows the avoidance of the burden of accurate dedicated segmentation and feature extraction methods prior to the classification [11].

DL tries to learn representations from input images using multilayer Neural Networks (NN), which enable us to obtain specific end outcomes such as segmentation or classification without hand-crafted features. In this work, two different NNs were designed, trained, validated and tested: one Segmentation Neural Network (SNN) for image segmentation and one Convolutional Neural Network (CNN) for image classification. The former had the aim to segment the whole-slide PB smears and the latter to classify individual erythrocytes regarding whether they are parasitized or not.

Therefore, by concatenating both networks with an intermediate step that prepares the output of the first one to input the second one, the main goal of this work is to design a NN-based system for the automatic detection of malaria. As a Digital Pathology System (DPS), it is devised to support work in clinical laboratories and give assistance in order to make quicker, less subjective decisions toward such diagnosis. The novel pipeline presented in this work shows a global segmentation accuracy for RBCs of 93.72% and a specificity for malaria detection of 87.04%.

This paper first summarizes the relevant related work; Section 2 describes the datasets used in the study as well as the required software and hardware; Section 3 describes each of the pipeline steps, detailing the architecture and training procedures of each NN; Section 4 lists the results for each stage of the pipeline; and Section 5 discusses the results, their issues and implications, and suggests future work.

### Related Work

Loddo et al. [9] review methods that have been proposed for automatic malaria diagnosis. Each of the works reviewed is analyzed for the typical workflow in image analysis: preprocessing, segmentation, feature extraction and classification. The pipeline proposed in our work does not require preprocessing nor manual extraction of features due to the use of DL NNs, therefore avoiding one of the most tedious steps of ML.

Most RBC segmentation methods use thresholding, morphological operations and marker-controlled watershed [9,11]. Tran et al. [12] used SegNet SNN [13] to segment RBCs, WBCs and the background, rendering single class accuracies of 91%, 95% and 81%, respectively. Comparatively, the segmentation step we present in this work shows higher single class accuracies for RBCs (93%) and non-erythrocyte (96%) using a SNN trained from scratch. Moreover, it does not recognize a third class. As discussed in Section 5, this implies a high rate of misclassification of WBCs and platelets as RBCs, therefore inducing errors in the next two stages of the pipeline.

Every work in reviewed in [9] has different scopes of classification: 60% (18/30) of the works classify the cells into two classes (infected/non-infected); two other works did not only classify the RBCs into these two classes, but also into lifecycle stages for species. All of the works report an accuracy and/or sensitivity higher than 70%, with only one sensitivity below 80%. The rest of the works distinguish infected cells into the species of the parasite, some including lifecycle stages. Their accuracy and/or sensitivity is lower than the two-class classification but also higher than 70%. Most of the studies based their classification on the extracted textural and geometric features [9].

On the other hand, Rajaraman et al. [11] evaluate the performance of pre-trained CNNs for the binary classification RBCs into malaria infected or non-infected. They conclude that they are a promising tool for feature extraction for this purpose, ResNet-50 being the one that outperformed the other candidates.

## 2. Materials

During the daily work in the Core Laboratory at the Hospital Clínic of Barcelona, digital images of PB smears stained with MGG were acquired. For this study, 517 images (RGB, 2400 × 1800 pixels) were obtained using the clinical microscope BX43 (Olympus, Tokyo, Japan) with 1000× magnification and the microscope digital camera DP73 (Olympus, Tokyo, Japan). These images were split into two datasets regarding whether they were infected by *Plasmodium* parasites (dataset B, 331 smears images, 19.7% of erythrocytes parasitized) or not (dataset A, 186 smears images). A and B are part of the dataset used in a recent publications by the authors of [14]. The images were used with the approval of the Research Ethics Committee of Hospital Clínic of Barcelona (HCB/2018/0322) and the datasets are available in [15] and [16].

Additionally, a third dataset (dataset C) was downloaded from the online repository Malaria Dataset (https://ceb.nlm.nih.gov/repositories/malaria-datasets/), which belongs to the Lister Hill National Center for Biomedical Communications (LHNCBC). It consists of 27,558 images of MGG stained masked and cropped individual erythrocytes (50% healthy, 50% *Plasmodium* parasitized). Such images are RGB and their dimensions range from 55 × 40 to 364 × 340 pixels. The three datasets are summarized in Table 1.

As per the computational side, the network architectures were designed, trained, validated and tested using MATLAB^®^ Deep Learning Toolbox^TM^ and a Nvidia Titan XP Graphics Processing Unit (GPU).

## 3. Methods

**Figure 2:**
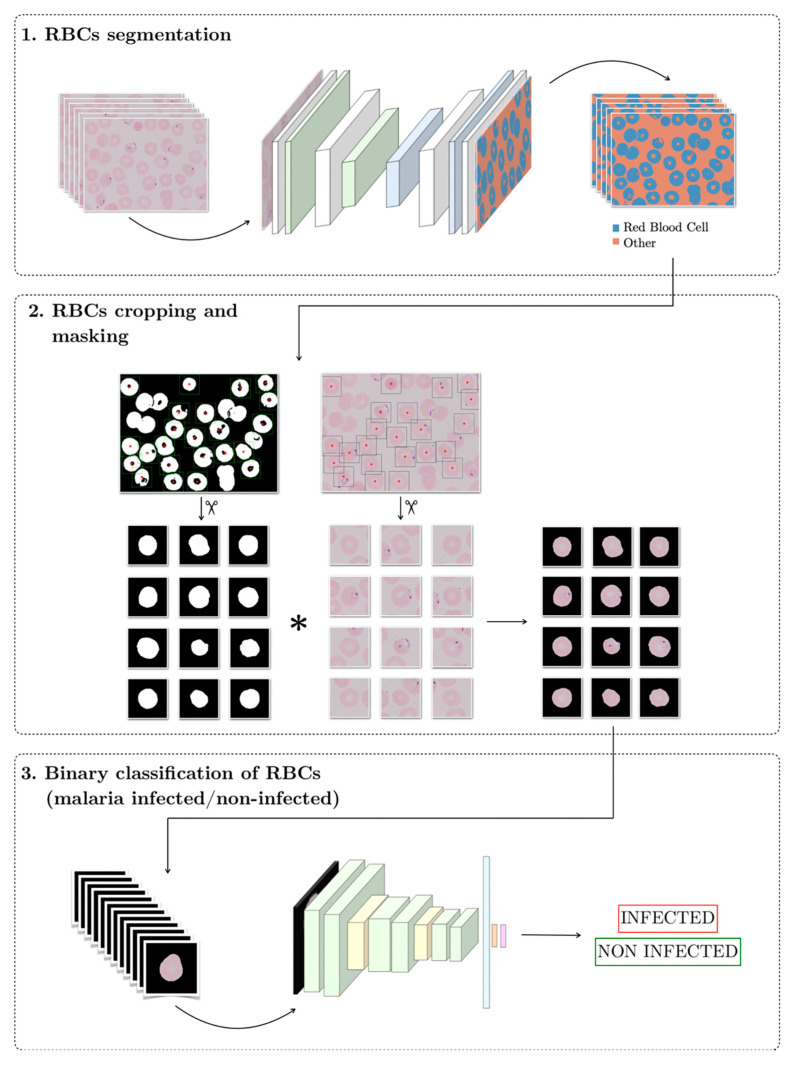
Scheme of the proposed solution pipeline.

The target of this work is to design a Clinical Decision Support System (CDSS) for malaria detection using neural networks. Figure 2 shows the structure of the pipeline, which consists of three stages in series. The input to the system is a peripheral blood smear digital image and the output should be the label for each individual erythrocyte, infected or non-infected. The code of the pipeline is available in Supplementary Materials.

### 3.1. RBC Segmentation

At this stage, a Segmentation Neural Network segments the RBCs of the input PB smear digital images by labeling each pixel as “RBC” if it belongs to an erythrocyte or as “other” if not.

#### 3.1.1. Network Design

**Figure 3:**
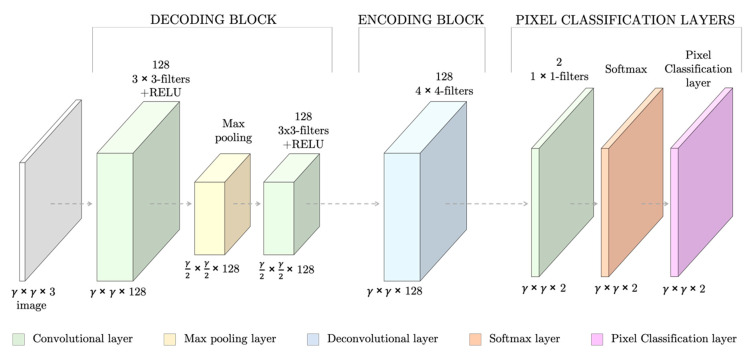
Design of the Segmentation Neural Network (SNN) for red blood cell (RBC) segmentation for an input RGB images of γ × γ spatial dimensions. At the bottom of each layer, the dimensions of its outputs are stated.

The SNN had seven fully convolutional layers, which summed 151,171 weights to train (Figure 3). The layers followed the specific architecture of SNNs [17]: the first layers (the decoding block) consisted of two sets of convolutional and RELU layers and a max pooling layer to down-sample the image to capture semantic/contextual information. They were then followed by a second block of up-sampling and deconvolutional layers (encoding block) to resize the output image to the input dimensions and filter at the same time. Lastly, a set of pixel classification layers enabled the individual labeling of the pixels.

#### 3.1.2. Dataset Creation

**Figure 4:**
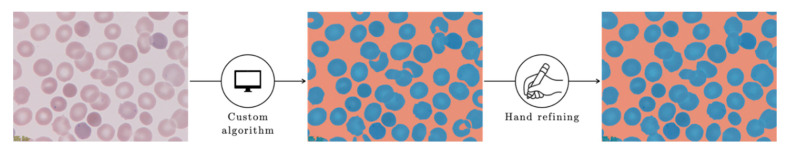
Pixel labeling of the smears into “RBC” (blue) or “other” (red). To speed up the manual labeling process, an algorithm was created to obtain a preliminary coarse label image which was then hand refined to obtain the ground truth labeling.

To train, validate and test the SNN, each of the 803,520,000 pixels from dataset A was semi-automatically labeled by applying a custom algorithm (Appendix A) followed by hand refining to obtain the ground truth labeling (Figure 4).

**Figure 5:**
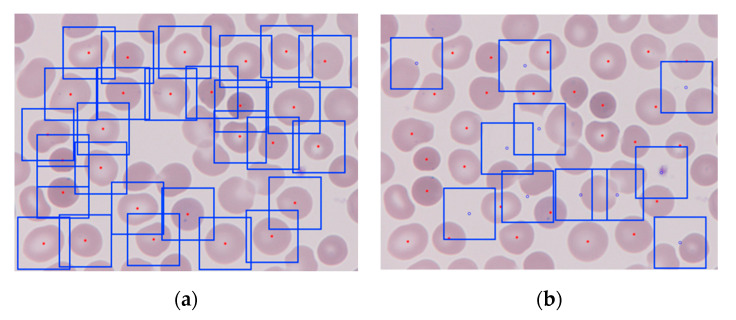
The 361 by 361 pixels patches generated from a PB wide field smear digital image: (**a**) erythrocyte-centered patches, the centroid is marked in red and the square boxes are each of the patches; (**b**) non-erythrocyte centered patches, centered at the blue dots.

Samples from five different patients constituted the dataset. One patient was randomly selected to be the test set (17 smear images) and the remaining 169 PB smears images were kept for training and validation. A greater number of images are required to train a NN. Therefore, 5911 square patches of 361 by 361 pixels were cropped from the wide field smear images to train and validate the network, i.e., in Figure 3, γ = 361. By analyzing the ground truth binary label masks, two types of patches were generated: (1) patches with and (2) patches without an erythrocyte at the center (Figure 5).

To crop the patches, the centroid coordinates of all the objects in the label image of value 1 (RBCs) were located. Those corresponding to individual RBCs were selected in terms of area and eccentricity, to exclude overlapping erythrocytes—dismissed also in clinical routine. Eccentricity was defined as a value in a range between 0 and 1 that corresponded to the ratio of the distance between the foci of the ellipse and its major axis length.

The following opening of the image allowed us to exclude small clusters that could remain after the hand refining step of the labeling process. As the centroid coordinates coincided in the label mask and the origin smear image, patches centered in such coordinates of width and height 361 pixels were cropped from it.

On the other hand, centers of the patches without any RBC in the center depend on the cells locations and were randomly generated in such a way that they were:

- Further than 180 pixels from any erythrocyte centroid, so they were different enough from erythrocyte-centered patches;
- Far apart enough from themselves, so they were all different and representative of the wide smear;
- Possible to crop, i.e., in such a distance from the borders that a patch of the desired dimensions could be generated.

It was desirable to obtain the same number of patches with and without an erythrocyte in the center. However, that was sometimes not possible, especially when the number of RBCs in the image was higher. In total, 3099 patches had an erythrocyte in the center while 2812 did not.

The SNN was trained and validated using the patches: the patches from the raw image paired with the patches with the expected output, and the corresponding ground truth labeling. As these patches had their origin in samples from four different patients, each patient’s set of patches was randomly split into the training (80%) and validation (20%) sets. Each patient’s patches were individually split to ensure that the final training and validation sets were composed by a combination of the four patients. Finally, all patients’ training sets were stacked together and shuffled, as well as the validation sets.

#### 3.1.3. Network Training and Validation

During the training stage, the neural network labels the pixels of the training images based on the features extracted. After each image is labeled, the difference between the ground truth segmentation and what the network predicted is measured using a loss function [18]. For this case, the loss function was the cross-entropy function for *k* mutually exclusive classes, with *k* = 2. To minimize this loss, stochastic gradient descent was used as the local optimization method with a learning rate μ = 0.001. The learning rate determines the step size toward the minimum.

The network went through the whole dataset 75 times, by passing through 64 training images at a time and the accuracy metrics on the validation data were calculated every 50 iterations.

#### 3.1.4. Network Testing

To test the network, the 17 wide field smear images that were kept as the test set were used. The SNN was fed with the wide field images, i.e., they were not cropped, and the accuracy was evaluated using the following metrics: single class mean accuracy, global accuracy and the Jaccard similarity coefficient.

### 3.2. RBCs Cropping and Masking

**Figure 6:**
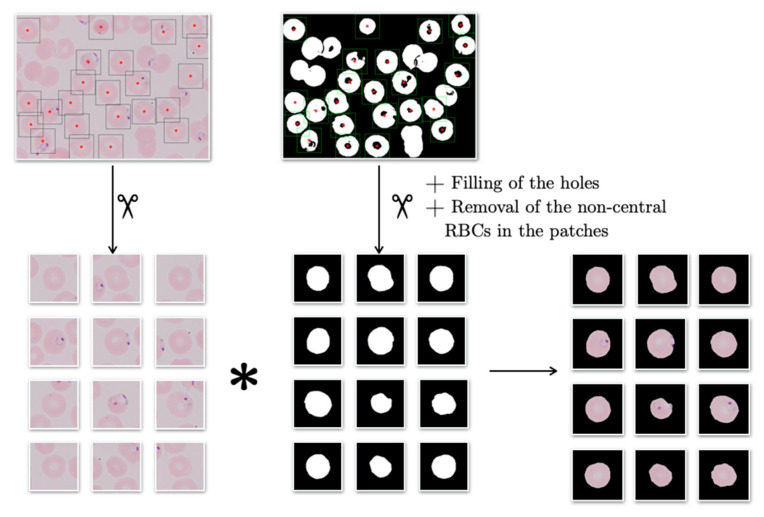
Process of cropping and masking individual RBCs. The image shown is from dataset B.

**Figure 7:**
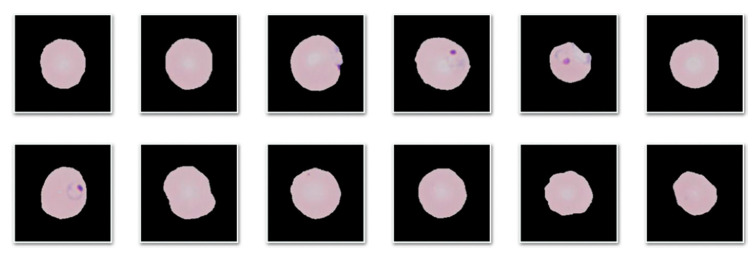
Cropped and masked patches resulting from the second step of the pipeline.

The purpose of this second step is to prepare the outputs from the SNN above to input the CNN in the third step. To do so, the individual erythrocytes of the labeled wide smear images exiting step 1 have to be cropped and masked. To crop them, the same process used to create the patches for the training and validation datasets in Section 3.1.2 was used: analyzing the binary label masks generated by the SNN in step 1; however, only generating erythrocyte centered patches. These patches were used to test the CNN in the third step and to resemble the training and validation sets they were masked. The masking algorithm dealt with the origin image patch and the corresponding binary label patch, in the manner schematized in Figure 6. The output of the algorithm was the element-by-element product of the origin patch and the labeled patch (Figure 7).

### 3.3. Binary Classification of RBCs

This last step aims to determine whether the RBCs cropped at the step above are *Plasmodium* parasitized or not. To do so, a Convolutional Neural Network was trained, validated and tested.

#### 3.3.1. Network Design

**Figure 8:**
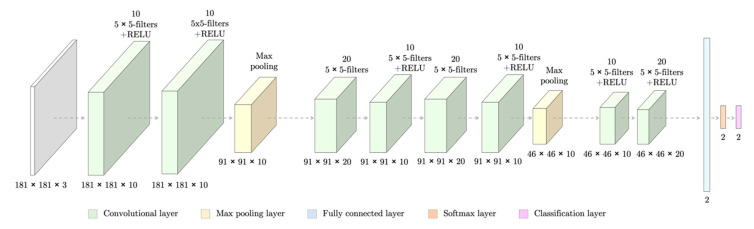
Design of a Convolutional Neural Network (CNN) able to classify erythrocytes patches of 181 × 181 pixels regarding whether they are infected by malaria or not. At the bottom of each layer, the dimensions of its outputs are stated.

The CNN had 13 layers and 30,758 weights to train (Figure 8). However, it did not have an encoding block as it was for classification purposes rather than segmentation. The network took as input patches of dimensions 181 × 181 pixels and gave as an output the probabilities of it belong to each of the classes (infected/non-infected).

#### 3.3.2. Datasets

The SNN designed in Section 3.1.1 was fed with the wide field images from dataset B (331 PB smears images with the diagnosis of malaria). Then, its erythrocytes were cropped with the process described in Section 3.2. The resulting patches were manually classified as infected (763) or non-infected (3116). These amounts were unsuitable to generate the training, validation and test sets as (1) there are not enough patches and (2) they are clearly unbalanced—4 non-infected RBCs per each infected erythrocyte. The unbalancing is expected, as normally in a wide field smear image, only a few erythrocytes are infected. However, dataset B was not balanced as it would have been reduced to 1526 images only.

The CNN had to be trained and validated with dataset C (27,558 images of masked RBCs, 50% *Plasmodium* parasitized), downloaded from the online repository Malaria dataset (https://ceb.nlm.nih.gov/repositories/malaria-datasets/) and tested with the labeled patches from dataset B. Dataset C was split 80% for training, 20% for validation.

#### 3.3.3. Network Training and Validation

This CNN performs similarly to the SNN for segmenting RBCs, but it classifies the whole patch instead of its pixels based on the features extracted. The loss function to evaluate was also the cross-entropy function for two mutually exclusive cases and optimized using the local optimization method of stochastic gradient descent with the learning rate μ = 0.01. It went through the whole dataset 30 times by batches of 128 patches. The accuracy metrics for the validation sets were acquired every 100 iterations.

#### 3.3.4. Network Testing

The CNN was tested using the patches that we generated from dataset B. By means of comparing these predicted classes to the true ones, the number of True Positives (TP), True Negatives (TN), False Positives (FP) and False Negatives (FN) were acquired. With these parameters, the confusion matrix could be built, and the statistical measurements of accuracy (1), sensitivity (2), specificity (3), positive predictive value (PPV) (4), and negative predictive value (NPV) (5) were calculated:

(1) $$\mathrm{Accuracy}=\frac{\mathrm{TP}+\mathrm{TN}}{\mathrm{TP}+\mathrm{FP}+\mathrm{TN}+\mathrm{FN}}$$

(2) $$\mathrm{Sensitivity}=\frac{\mathrm{TP}}{\mathrm{TP}+\mathrm{FN}}$$

(3) $$\mathrm{Specificity}=\frac{\mathrm{TN}}{\mathrm{TN}+\mathrm{FP}}$$

(4) $$\mathrm{PPV}=\frac{\mathrm{TP}}{\mathrm{TP}+\mathrm{FP}}$$

(5) $$\mathrm{NPV}=\frac{\mathrm{TN}}{\mathrm{TN}+\mathrm{FN}}$$

As shown in [19], the network was tested using two different infected probability cut-offs (0.3 and 0.5), but, given the small variation, the usual cut-off of 0.5 was maintained.

## 4. Results

### 4.1. RBC Segmentation

**Figure 9:**
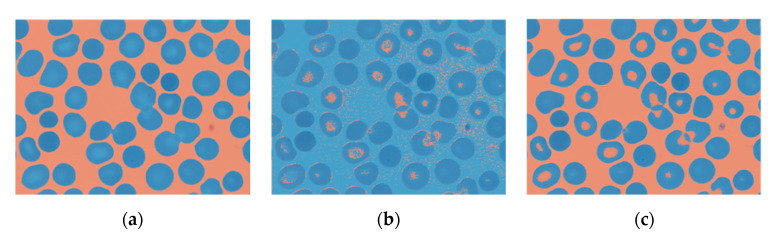
In blue, the pixels labeled as “erythrocyte”. They appear overestimated by the SNN predictions when trained with erythrocyte centered patches only. (**a**) Ground truth segmentation, (**b**) SNN segmentation prediction when trained with erythrocyte centered patches only, and (**c**) SNN segmentation prediction when trained with erythrocyte centered and non-erythrocyte centered patches.

For the first classification attempt, the SNN was trained using only the erythrocyte centered patches and their equivalent label images, resized to 64 × 64 pixels. However, when the SNN was fed with the wide field smears of the test set, the resulting segmentation overestimated the pixels classified as RBCs (Figure 9b). The next step was to generate patches that did not have an erythrocyte in the center, with two purposes: (1) to have the recurrence of pixels of both classes balanced, and (2) to train the network with images representative of the whole smear.

In this second step, the network was trained, validated and tested with the 361 × 361 pixels erythrocyte centered and non-centered patches and the corresponding label images resized to 8 × 8, 16 × 16, 32 × 32, 64 × 64, and 128 × 128 pixels. The test set global accuracies for all of them was within the interval of 90.29% and 93.72%; the highest one corresponded to the training set with the largest patches. The elapsed training time grew exponentially, with a minimum of 7 min 47 s (patches of 8 × 8 pixels) and a maximum of 44 min and 22 s (patches of 128 × 128 pixels). As the SNN had to be trained only once, the one that had been trained with the largest patches was selected, as it provided higher accuracy. This approach allowed for higher single class accuracies (Figure 9c).

**Figure 10:**
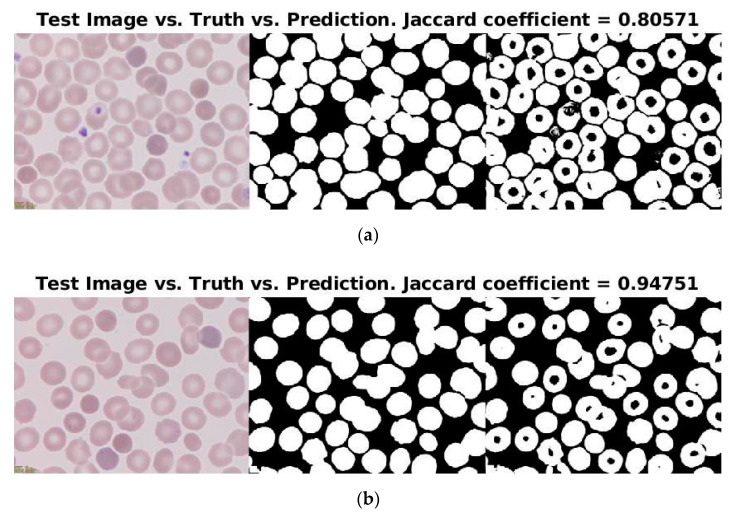
Predictions of the test set with (**a**) the lowest and (**b**) the highest Jaccard coefficients of similarity.

Moreover, 88% (15/17 smears) of the segmentations had a Jaccard coefficient larger than 0.85 with respect to the ground truth segmentation. Figure 10 displays the segmentations with the lowest (0.80571) and the highest (0.94751) Jaccard coefficients. Although both predictions were visually inspected as almost as good, it is observable that the biggest issue were the platelets; i.e., the higher the number of platelets, the lower the Jaccard coefficient, as they were wrongly labeled as RBCs. Additionally, both images had the same issue when the central areas of the erythrocytes, as they were predicted as “non-erythrocyte”.

### 4.2. RBCs Cropping and Masking

**Figure 11:**
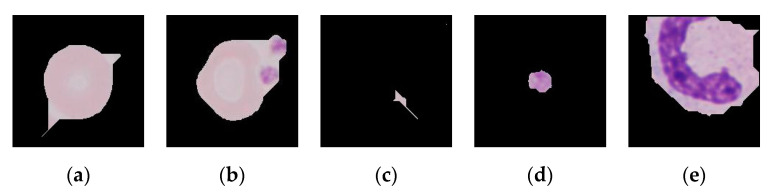
The cropping and masking of the erythrocytes gave some recurring problems that could be summed up in these five types: (**a**) masked RBCs with pointy appearance, (**b**) masked RBCs fused with platelets, (**c**) patches with small remains of undefined shape, (**d**) patches with a platelet as the centered object instead of a RBC, (**e**) masked WBCs.

Despite the fact that most of the patches looked as desired (Figure 6), some unwanted patches recurrently appeared (Figure 11):

### 4.3. Binary Classification of RBCs

It took 61 min and 14 s for the CNN to be trained and validated. The final accuracy on the validation set was 95.00%, whereas for the test set it was 75.39%. However, accuracy over the test set was not a good indicator of the performance considering the disproportion of the classes in such set: four non-infected RBCs per every parasitized erythrocyte. In other words, assuming that the performance of the classifier had rendered a total of true positives (TP) equal to zero, the accuracy would have already been 72.38%. Therefore, sensitivity (17.90%), specificity (87.04%), PPV (22.85%) and NPV (83.96%) were calculated.

### 4.4. Pipeline Execution

The whole pipeline was run in ten different patients’ blood smear images (five infected, five non-infected). Each patient had an average of 52 ± 30 images and the average time they took to go through the pipeline was 48.9 ± 25.1 s, 0.8 ± 0.2 s per image. After this time, the patches the network suspected to be infected were shown to the pathologists, so they made the final decision.

## 5. Discussion

The automation of malaria detection in PB smear digital images has often been addressed within the usual ML framework: preprocessing, segmentation, feature extraction and classification [9]. This work presents a novel design of a three-stage pipeline for the same purpose which does not require preprocessing nor the manual extraction of features due to the use of deep learning NNs. The aim of the pipeline presented is to segment whole cells from the background in the first level with an SNN and then to detect the parasites inside the cells with a CNN. This two-level approach gives the system the flexibility to be used for other digital pathology (DP) purposes by adapting and reusing the stages. For instance, the SNN could be extended to other clinical practices as RBC segmentation is also the first step for various clinical studies, as cell counting or cell shape identification are key for the diagnosis of red cell diseases like sickle cell anemia [20]. Furthermore, as Molina et al. published in [14], it is important to differentiate malaria parasites and other RBC inclusions, such as Howell–Jolly bodies and Pappenheimer bodies, and the CNN in the third stage could be retrained to detect them.

Moreover, the pipeline runs through each patient’s digital smears in less than 50 s, spending less than a second on each image in average. Then, the pathologists need to analyze only the patches the CNN classified as potentially infected to determine whether they have malaria or not. This is a major outcome considering that the average time pathologists spend analyzing patients’ PB smears ranges between 30 and 60 min per patient [5].

### 5.1. RBC Segmentation

The SNN for RBC segmentation rendered a high accuracy when trained with both erythrocyte-centered and non-erythrocyte-centered patches, showing that this was a feasible approach for data augmentation. The methods used in learning-based cell image segmentation to obtain the training patches are commonly (a) a sliding window with the same size that crops the film like a grid, or (b) the ground truth bounding box method [21]—see Li et al. [22]. The ground truth bounding box method is what we used in this work when the erythrocyte-centered patches were created. Nevertheless, more patches were created with no cells in the center, which implies more data available and rendered a better result, as shown in Section 4.1.

While most RBC segmentation methods are based on thresholding and/or morphological operations [9], some other works have started using deep learning approaches. Behind such approaches is the idea to overcome the dependency on acquisition conditions of the peripheral blood smear images [12] as brightness or color [9], thus avoiding preprocessing. Tran et al. [12] reported single class accuracy of 91% for erythrocytes and 81% for the background. They also included a third class, white blood cells (WBCs), with a single class accuracy of 95%. Our work shows higher single class accuracies for both erythrocytes (93%) and the background (96%). It is remarkable that the SNN in our work was trained from scratch and had many fewer parameters to train than the one that Tran et al. [12] used, the pretrained SegNet [13].

However, the segmentation rendered by the SNN in our work had two main drawbacks: (1) the labeling of the central part of the RBCs as non-erythrocyte (FN), and (2) the labeling of the platelets and WBCs as erythrocytes (FP). These problems are potentially driven by the similarity of tones: the color of the central areas of the RBCs is similar to the background and platelets and WBCs; despite not being the same as the RBCs, it is more similar to them than to the light background. Differences in sizes of RBCs, platelets and WBCs do not reduce the error as SNNs are scale independent. These drawbacks limited the performance of the subsequent steps.

Future work should incorporate the annotation of more classes in the training set (e.g., RBCs, platelets, WBC and background) so that the SNN differentiates them, improving segmentation.

### 5.2. RBCs Cropping and Masking

The main limitation of this step is its dependence on the step above, which has the two aforementioned drawbacks. While the labeling of the central part of the RBCs as non-erythrocyte could be overcome by the closing of the image, the labeling of the platelets and WBCs as erythrocytes could not be completely solved. Some platelets and remaining small clusters (dirt, debris) were removed by opening the image but some remained, as well as the WBCs. Opening the images with larger radii would have caused the removal of RBCs.

There exist in the literature more complex cell cropping algorithms that could benefit this second step of the pipeline. While these approaches would not benefit from the segmentation step before, they would avoid the transmission of errors into the third step and could also potentially include individual erythrocytes extracted from overlapping RBCs. For instance, Xu et al. [21] locate cells in the smear by entropy evaluation and Al-Hafiz et al. [23] segment RBCs in the smears by using boundary-based methods to automatically compute the threshold. Improvement in this second step should also translate into better classification results in the next step. Additionally, these approaches could also benefit the algorithm developed to help with the hand labeling of dataset A (Appendix A).

### 5.3. Binary Classification of RBCs

The CNN proved to discriminate parasitized from healthy RBCs, as the accuracy for the validation set was 95%. However, it had no generalization power, and, when evaluated with the test set, the accuracy was much lower (75%). Accuracy was not a good indicator due to the limitation that implies the imbalance of the test set (1 parasitized erythrocyte per every 4 healthy RBCs). Comparing the performance of the CNN to the gold standard, the microscopic analysis of stained blood films: NPV (84%) and specificity (87%) are similar to the gold standard (90.40% and 92.20%, respectively), but PPV (23%) and sensitivity are not (18%), as in the gold standard they are 69.80% and 74.30% [24]. It should be taken into account that the training and validation sets had the same origin (Malaria dataset from the LHNCBC) and the test set did not as it was part of the collection of images of the research group [14,25] and too small to be used also for training and validation. From this fact arises some key limitations:

1. Acquisition parameters and resolutions are different for each dataset, as well as the cropping and masking algorithms.
2. All the images from the training and validation sets had originally different dimensions and proportions, i.e., they were not all square. To feed the network, they were resized and reshaped, inducing deformations to the set and consequent training errors to the network.
3. The test set was acquired using the previous two steps of the datasets, therefore the unwanted patches (Figure 11) were also given as inputs to the network. As platelets have the same color as the parasites, all the patches that were undesirably cropped platelets only were classified as false positives.

Although the goal was to be able to train, validate and test the network with self-acquired digital PB films images, more indicative results could have been obtained by mixing the patches from dataset B with the ones from the Malaria dataset (dataset C) and training, validating and testing the network with a mix of these. Future work should evaluate this proposal as well as try to generate a dataset (acquire and label) with enough data to do all the steps with it, a long process, or to perform data augmentation, especially to create more parasitized patches, less abundant in the blood smears. Not to alter the fact that they are patches with a centered cell and look realistic, operations that can be applied to the original patches are any rotation or reflection, moderate scaling, moderate change of brightness or adding moderate pixel noise. Moreover, the final step should be giving the CNN the power to distinguish between *Plasmodium* species and into lifecycle stages as some other works have already tried, either by annotating a broader training set with these labels or without the use of DL NNs [9].

## 6. Conclusions

The main contribution of this paper is a novel three-stage pipeline involving two neural networks, a SNN and a CNN, to detect malaria in digital images of whole-slide peripheral blood smears. Both networks were designed, trained, validated and tested from scratch. The three stages of the pipeline were: (1) red blood cell segmentation, (2) RBC cropping and masking, and (3) the binary classification of RBCs into infected or non-infected.

The performance of the first stage, RBC segmentation, achieved a global accuracy of 93.72%. The segmentation of cells is a key step in digital pathology systems and would allow the network to be used for other purposes, such as cell count or morphological studies of RBCs.

The CNN for binary classification of RBCs as infected or not rendered high accuracy values for the validation set but did not show a good generalization power for the test set. This work reveals the underlying reason for this and paves the way for future improvements.

## Figures and Tables

**Table 1:** Summary of the three datasets used to train, validate and test the pipeline.

Dataset	Number of Images	Dimensions
A	186 PB smears	2400 × 1800 pixels
B	331 PB smears with the diagnosis of malaria	2400 × 1800 pixels
C	27,558 masked red blood cells (50% *Plasmodium* parasitized)	From 55 × 40 to 364 × 360 pixels

**Figure A1:**
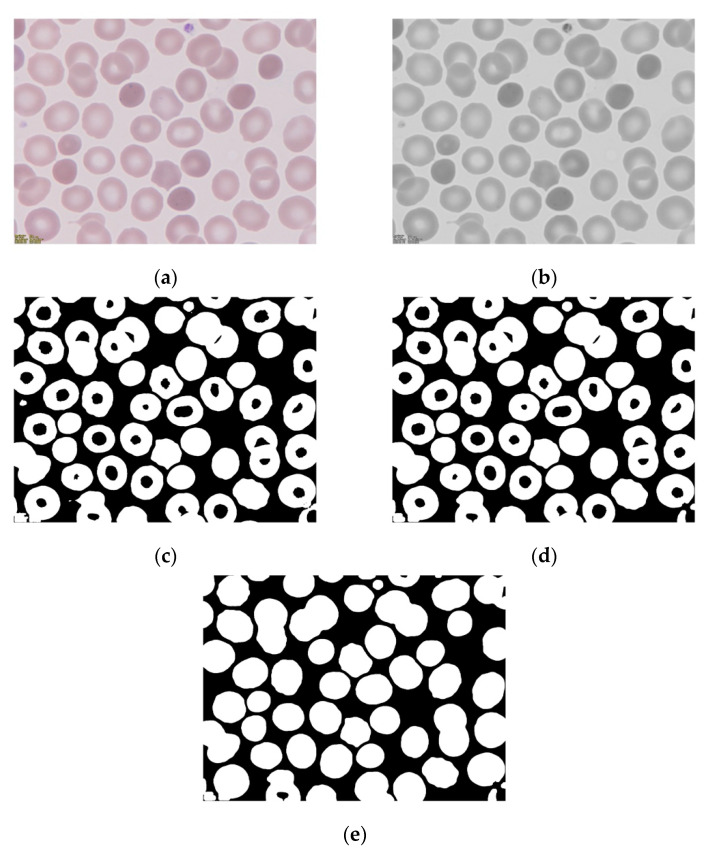
Steps performed by the semi-automation labeling algorithm to create a binary image of classes erythrocyte (1) and non-erythrocyte (0). Each panel illustrates the result of the steps: (**a**) origin image, (**b**) visualization of the green channel only, (**c**) image thresholding at intensity value 190, (**d**) opening of the image with a disk of radius 20 pixels, (**e**) filling of the image.

## Supplementary Materials

The source code for this work is available online through doi:10.5281/zenodo.3828050, 10.17632/c37wnbbd3c.1, 10.17632/2v6h4j48cx.1.

## Acronyms

CDSS	Clinical Decision Support System
CNN	Convolutional Neural Network
DL	Deep Learning
DP	Digital Pathology
DPS	Digital Pathology System
FN	False Negative
FP	False Positive
GPU	Graphics Processing Unit
MGG	May-Grünwald–Giemsa
ML	Machine Learning
NN	Neural Network
NPV	Negative Predictive Value
PB	Peripheral Blood
PPV	Positive Predictive Value
RBC	Red Blood Cell
RBG	Red-Green-Blue
SNN	Segmentation Neural Network
TN	True Negative
TP	True Positive
WBC	White Blood Cell
WHO	World Health Organization

## Appendix A

Before starting the labeling itself, the creation of a custom label automation algorithm was crucial to ease the tedious work of labeling every pixel in the images, as explained in Section 3.1.2. Such an algorithm was thought to create a labels to mask the image of the two categories for this classification: “erythrocyte” and “other” (i.e., non-erythrocyte). The algorithm applied thresholding and morphological operations on the green channel of the origin PB smear images. The output of the algorithm was thought to be a logical binary image that assigns 1 to the pixels belonging to an erythrocyte and 0 to other class. The sequence of procedures is shown in Figure A1:

1. The selection of the green channel of the RGB image to obtain a grayscale enhanced image. Most malaria detection works reviewed by Loddo et al. in [9] based their analysis on such a channel. The green channel in blood smear images renders a higher contrast between the structures, given that it is better at maintaining high frequency feature information than the other two channels [26].
2. The thresholding of the image at intensity value τ = 190.
3. The opening of the binary image with a disk of 20 pixels of radius.
4. The filling of the remaining holes.

The algorithm also included Gaussian filtering with sigma = 3 between steps one (Figure A1b) and two (Figure A1c). However, this had no significant effect and thus it was dismissed to simplify the procedure.

The search of the values of sigma for the thresholding and radius of the opening disk was not automated as they were visually fixed to suit the images of the five patients in dataset A. The aim was for the algorithm to render a preliminary coarse segmentation to ease the following manual refining. Therefore, an accurate, generalizable segmentation was not intended as this does not act in the pipeline; neither was it devised to replace the SNN but to speed up the creation of a dataset to train, validate and test it. The final algorithm had a global accuracy of 54.50% when compared to the ground truth label images obtained after the hand refining.
